# Effects of vitamin B-12 supplementation on neurologic and cognitive function in older people: a randomized controlled trial^1^,^2^

**DOI:** 10.3945/ajcn.115.110775

**Published:** 2015-07-01

**Authors:** Alan D Dangour, Elizabeth Allen, Robert Clarke, Diana Elbourne, Astrid E Fletcher, Louise Letley, Marcus Richards, Ken Whyte, Ricardo Uauy, Kerry Mills

**Affiliations:** 3 Faculty of Epidemiology and Population Health, London School of Hygiene & Tropical Medicine, London, United Kingdom;; 4 Clinical Trial Service Unit & Epidemiological Studies Unit, University of Oxford, Oxford, United Kingdom;; 5 Medical Research Council General Practice Research Framework, London, United Kingdom;; 6 Medical Research Council Unit for Lifelong Health and Ageing at University College London, London, United Kingdom; and; 7 Department of Clinical Neurosciences, King’s College, London, United Kingdom

**Keywords:** cognitive, neurologic, older people, vitamin B-12, peripheral and central nerve conduction

## Abstract

**Background:** Moderate vitamin B-12 deficiency is relatively common in older people. However, there is little robust evidence on the effect of vitamin B-12 supplementation on neurologic and cognitive outcomes in later life.

**Objective:** We investigated whether vitamin B-12 supplementation benefits neurologic and cognitive function in moderately vitamin B-12–deficient older people.

**Design:** We conducted a double-blind, randomized, placebo-controlled trial in 7 general practices in South East England, United Kingdom. Study participants were aged ≥75 y and had moderate vitamin B-12 deficiency (serum vitamin B-12 concentrations: 107–210 pmol/L) in the absence of anemia and received 1 mg crystalline vitamin B-12 or a matching placebo as a daily oral tablet for 12 mo. Peripheral motor and sensory nerve conduction, central motor conduction, a clinical neurologic examination, and cognitive function were assessed before and after treatment.

**Results:** A total of 201 participants were enrolled in the trial, and 191 subjects provided outcome data. Compared with baseline, allocation to vitamin B-12 was associated with a 177% increase in serum concentration of vitamin B-12 (641 compared with 231 pmol/L), a 331% increase in serum holotranscobalamin (240 compared with 56 pmol/L), and 17% lower serum homocysteine (14.2 compared with 17.1 μmol/L). In intention-to-treat analysis of covariance models, with adjustment for baseline neurologic function, there was no evidence of an effect of supplementation on the primary outcome of the posterior tibial compound muscle action potential amplitude at 12 mo (mean difference: −0.2 mV; 95% CI: –0.8, 0.3 mV). There was also no evidence of an effect on any secondary peripheral nerve or central motor function outcome, or on cognitive function or clinical examination.

**Conclusion:** Results of the trial do not support the hypothesis that the correction of moderate vitamin B-12 deficiency, in the absence of anemia and of neurologic and cognitive signs or symptoms, has beneficial effects on neurologic or cognitive function in later life. This trial was registered at www.isrctn.com as ISRCTN54195799.

See corresponding editorial on page 529.

## INTRODUCTION

Vitamin B-12 deficiency, which is frequently attributable to age-related gastric atrophy, is relatively common in later life and affects about one-sixth of people aged >75 y in the United Kingdom (1). Vitamin B-12 is required for the methylation of myelin, neurotransmitters, and membrane phospholipids and is essential for the integrity of the central and peripheral nervous systems (2, 3). Severe deficiency of vitamin B-12 typically presents as sensory disturbances in the extremities (tingling and numbness) and loss of vibration and joint position sense, together with motor problems and abnormalities of gait, impaired cognition, and depression (2, 3). Neurologic and cognitive manifestations of severe vitamin B-12 deficiency are largely responsive to treatment with vitamin B-12 (repeated intramuscular injection), although improvement may take time (4), and the severity and duration of neurologic abnormalities influence the degree of recovery (2, 3).

Neurologic, cognitive, and psychological abnormalities also occur in individuals with moderate vitamin B-12 deficiency (serum vitamin B-12 concentrations: 107–210 pmol/L) (5), although the evidence of a direct association between vitamin B-12 status and neurologic function (6–9) or cognitive function (10, 11) has been mixed. Oral supplementation with crystalline vitamin B-12 is routinely used to correct hematologic variables of moderate deficiency (2, 3, 12), but to our knowledge, there has been no previous evidence on the efficacy of treatment of neurologic or cognitive function in older people with moderate vitamin B-12 deficiency (2, 3).

The aim of the current trial was to determine whether daily supplementation for 12 mo with 1 mg vitamin B-12 or a placebo in older people with moderate vitamin B-12 deficiency in the absence of anemia would have beneficial effects on peripheral and central neurologic function and on cognitive function.

## METHODS

### Participants

Details of the trial protocol have been published (www.isrctn.com; ISRCTN54195799) (13). Participants aged ≥75 y were enrolled at 7 general practices in South East England that were members of the Medical Research Council General Practice Research Framework or the National Institute of Health Research Primary Care Research Network. Potentially eligible participants were identified by a computer search after the exclusion of individuals with diabetes, dementia, or epilepsy. An additional manual check of health records was carried out by trained nurses to exclude individuals with alcohol addiction, pacemakers, or other implanted metallic devices (for whom central neurophysiologic testing was contraindicated), residents of nursing homes, and anyone with a previous diagnosis of pernicious anemia. After confirmation by their general practitioners, eligible individuals were invited by mail to participate in the trial. Individuals who reported current consumption of vitamin B-12 supplements or who had received a vitamin B-12 injection in the previous 6 mo were excluded. Interested eligible participants were invited to attend their general practices for a screening appointment where research nurses clarified any queries and administered the Mini-Mental State Examination (14) to exclude significant cognitive impairment. Participants with a Mini-Mental State Examination score ≥24 (maximum score: 30) were asked to provide a blood sample to assess serum vitamin B-12 and hemoglobin concentrations. Individuals with very-low vitamin B-12 concentrations (<107 pmol/L, which is a cutoff typically used for deficiency; Beckman Coulter assay, Beckman Coulter Inc.) or who were shown to have anemia (hemoglobin concentration <110 g/L for women and <120 g/L for men) were excluded and referred to their general practitioners for additional assessment. Individuals with moderate vitamin B-12 deficiency who did not have anemia (serum vitamin B-12 concentrations ≥107 and <210 pmol/L [Beckman Coulter assay (1)] and hemoglobin concentrations ≥110 g/L for women and ≥120 g/L for men) were eligible to join the trial and were invited to attend a baseline appointment at King’s College Hospital, London.

### Procedures

At the baseline appointment, the study manager discussed the trial with potential participants and obtained written informed consent before random treatment allocation. Allocation codes were obtained from a central computerized randomization service. Allocation to treatment was balanced by age (75–79 and ≥80 y) and sex. Allocated treatment consisted of a single tablet administered daily that was identical in size, shape, color, smell, and taste for both the intervention and placebo and packaged into identical pots each of which contained 70 tablets. Each intervention tablet contained 1 mg vitamin B-12 (cyanocobalamin). The dose was selected to be greater than the minimum Recommended Daily Intake (2.5 μg) required to correct vitamin B-12 deficiency in older people (15) and is safe (16) (there is no defined dietary intake upper limit for vitamin B-12). Because ∼1–2% of an oral dose of vitamin B-12 is absorbed (by passive diffusion), this dose would be expected to provide 10–20 μg/d in the absence of an intrinsic factor required for active absorption (12). All study personnel were blinded to the treatment allocation.

Before the baseline appointment, participants were invited to complete a postal questionnaire that was used to collect information on diet and alcohol consumption. Psychological health was also assessed by postal questionnaire at baseline and 12 mo with the 30-item General Health Questionnaire (17). At the baseline appointment, data were collected on educational history and history of previous stroke or myocardial infarction. Data were also collected on current prescribed medication. At baseline and 12 mo after random assignment, height (to the nearest 0.1 cm) and weight (to the nearest 0.1 kg) were measured, and the timed up-and-go test (18) was administered to assess mobility.

### Assessment of neurologic function

At baseline and after 12 mo, a single physician (KM) assessed clinical measures of neurologic function (presence or absence of knee and ankle jerks and of joint position sense and vibration sense in the great toe) and conducted a standard battery of peripheral nerve-conduction tests (including motor and sensory nerve conduction in the right superficial peroneal, sural, common peroneal, and tibial nerves), and central motor conduction tests. Skin temperature of the dorsum of the foot was measured, which allowed for correction for temperature differences between first and second visits. The sensory action potential (SAP)^8^ amplitude (maximum deviation of the electrical response) and conduction velocity (distance divided by onset latency) were measured. Common peroneal, tibial, and ulnar motor conduction were measured by recording from extensor digitorum brevis, abductor hallucis (AH), and abductor digiti minimi (ADM) muscles, respectively. Nerves were stimulated supramaximally at proximal and distal sites, and conduction velocity was calculated. Compound muscle action potential (CMAP) amplitude, distal motor latency, and F-wave latency (a measure of conduction time from the distal stimulation site to the spinal cord) were also measured.

Central motor conduction in the corticospinal tract was measured by using transcranial magnetic stimulation, which painlessly and noninvasively excites the motor cortex (19). A 13-cm diameter circular coil connected to a magnetic stimulator that provided a monophasic pulse was centered over the vertex to excite the hand area of the left motor cortex. The threshold for excitation was determined by using a standard technique (20). With the right ADM muscle partially activated voluntarily, 8 stimuli at 1.2 times the threshold were delivered to evoke motor evoked potentials (MEPs), the mean amplitude and minimal latency of which were measured. The time to response in a given muscle was subtracted from an estimate of the peripheral nerve conduction time to calculate the central motor conduction time. Similarly, by using a double cone coil, the leg area of motor cortex was excited to measure MEPs evoked in AHs. Each participant received a maximum of 70 brain stimuli. Any individuals shown to have significant neurologic deficit were referred to their general practitioners for additional assessment.

The primary outcome of the trial was the posterior tibial CMAP amplitude evoked by distal stimulation. The negative peak amplitude of the peripherally evoked CMAP reflects the number of motor axons that can be accessed by an electrical stimulus, which, in turn, reflects muscle strength (21, 22). Of 10 secondary neurologic outcomes, 3 outcomes assessed motor nerve conduction (posterior tibial conduction velocity, common peroneal CMAP amplitude, and common peroneal conduction velocity), 4 outcomes assessed sensory nerve conduction (sural SAP amplitude, sural conduction velocity, superficial peroneal SAP amplitude, and superficial peroneal conduction velocity), and 3 outcomes assessed central nerve conduction (mean right ADM MEP amplitude, central motor conduction time to the right ADM, and central motor conduction time to the right AH).

### Assessment of cognitive function

At baseline and after 12 mo, the study manager (KW) administered a range of cognitive function tests. In accordance with international guidance (23), the main cognitive outcome was a test of memory, i.e., a 16-item word list from the California Verbal Learning Test (CVLT) (24). The sum of words recalled after each of 3 repeats and the number of words recalled after a 20-min delay formed the main cognitive outcome. The identical version of the CVLT was used at baseline and after 12 mo. Other cognitive outcomes were as follows: processing speed assessed by using the oral version of the symbol letter modality test (25) for which the outcome was the number correct in 90 s; simple and choice reaction time (26) assessed by using an electronic reaction timer that provided 20 single (simple reaction time) or 40 multiple (choice reaction time) challenges; and executive-function assessed by using a verbal fluency test (animal naming) over 60 s (27).

### Adherence to randomized intervention

Adherence to allocated treatment was measured by counting the number of tablets returned at the end of the study. At baseline and 12 mo after random assignment, blood samples were collected to measure serum concentrations of vitamin B-12 (microbiologic assay; CV range: 5–7%), holotranscobalamin (Axis-Shield radioimmunoassay; CV range: 5–8%; Axis-Shield plc), total homocysteine (Abbott IMx analyzer; CV range: 2–3%; Abbott Laboratories), and folate (chloramphenicol-resistant microbiologic assay; CV range: 5–8%). A full blood count was analyzed for hemoglobin. Baseline appointments were held, on average, a median of 63 d (IQR: 38–119 d) after the screening appointment. At baseline, vitamin B-12 was assessed by using a microbiologic assay (that estimated serum concentrations ∼25% higher than did the Beckman Coulter method used at the screening appointment), and confirmed that randomly assigned participants had low vitamin B-12 status at study entry (88% of subjects had vitamin B-12 status below the median value for the microbiologic assay) and did not have anemia. There were no preset criteria for participant withdrawal during the trial. Any participants who stopped taking the study medication were invited to attend their scheduled follow-up assessment at 12 mo.

### Statistical analysis

A sample size of 100 individuals for each allocated treatment group was selected that, with an assumed 30% dropout over 12 mo, would have given 90% power to detect a ≥28% change in the primary outcome of posterior tibial CMAP amplitude with 5% significance. Posterior tibial CMAP amplitude is a marker of foot muscle strength, and a 28% increase is likely to be associated with clinically relevant improvements in physical coordination and balance in older people (28, 29). The primary analysis was carried out on an intention-to-treat basis. The ANCOVA models were adjusted for baseline measures. Adjusted models further allowed for age and sex, and in the case of the primary outcome, we also adjusted for skin temperature. All models with continuous outcomes were boot strapped to allow for nonnormal distributions. ORs for binary variables were calculated by using logistic regression. Variables that were not normally distributed are presented as medians with IQRs. No subgroup analysis was prespecified. An independent data monitoring and safety committee assessed safety data. Results are presented as appropriate effect sizes with 95% CIs. This study was reviewed and approved by the National Research Ethics Committee (08/H0305/18) and the London School of Hygiene & Tropical Medicine Ethics Committee (no. 5298).

## RESULTS

### Study participants

**FIGURE 1:**
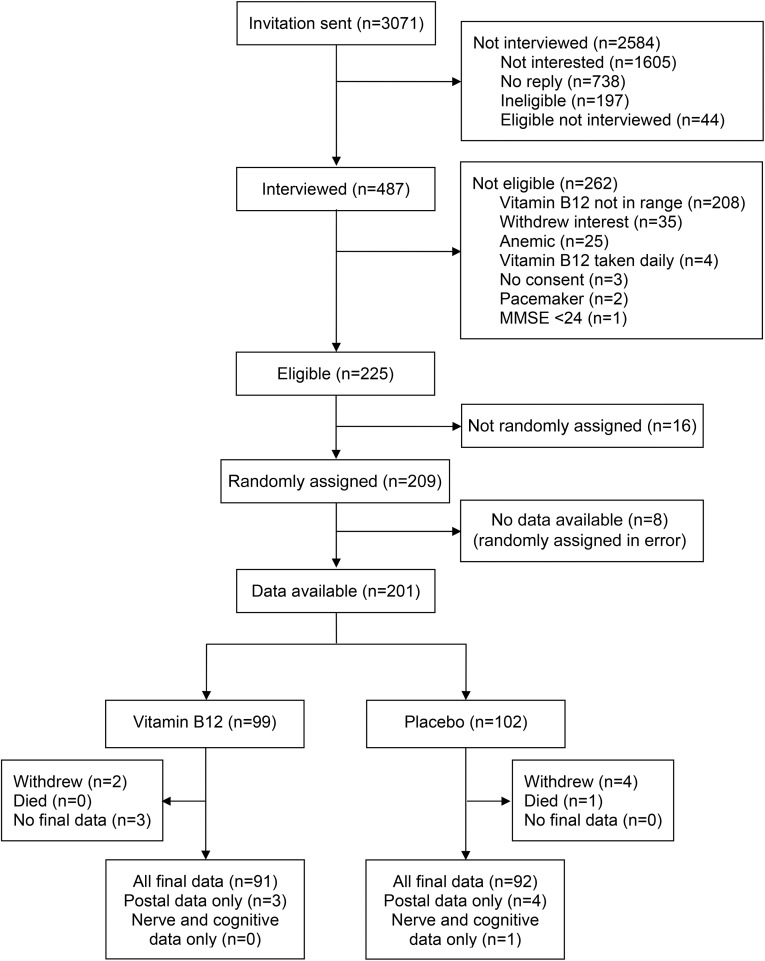
Consolidated Standards of Reporting Trial flowchart for the Older People and Enhanced Neurological Function study. MMSE, Mini-Mental State Examination.

Participants were screened between November 2008 and February 2010. Invitation letters were sent to 3071 potential participants, and 487 individuals (16%) agreed to attend a screening appointment (**Figure 1**). After screening, 262 potential participants were shown to be ineligible, largely because their serum vitamin B-12 concentrations were out of range. Of 209 participants who were randomly allocated to the study between January 2009 and May 2010, 8 subjects were randomly assigned in error (as a result of protocol deviations) and provided no additional data. Valid data at baseline were available on 201 participants. Six participants withdrew from the study (2 subjects from the vitamin B-12 arm and 4 subjects from the placebo arm), and one participant died. There were no other reported serious adverse events. Three individuals who continued study medication (all allocated to vitamin B-12) did not provide any data at 12 mo. Outcome data on 191 participants (95% of randomly assigned subjects) were available after 12 mo of intervention. At baseline, sociodemographic variables, medical history, use of prescribed medication, diet, and serum B-vitamin status were similar between allocated groups (**Table 1**). The study arms were well matched at baseline for neurologic (**Table 2**), cognitive, and psychological (**Table 3**) function outcomes.

**TABLE 1:** Baseline characteristics of OPEN study participants by allocated treatment^1^

	Vitamin B-12	Placebo
Participants, *n*	99	102
Sex, M, *n* (%)	46 (46.5)	48 (47.1)
Age		
Age, y	79.9 ± 3.5^2^	80.1 ± 3.7
75–79, *n* (%)	56 (56.6)	57 (55.9)
≥80, *n* (%)	43 (43.4)	45 (44.1)
Education		
Age at leaving, y	18.3 ± 5.3	17.8 ± 6.6
No qualifications, *n* (%)	21 (21.4)	33 (33.0)
Basic or clerical, *n* (%)	16 (16.3)	18 (18.0)
Advanced or university, *n* (%)	33 (33.7)	19 (19.0)
Other, *n* (%)	28 (28.6)	30 (30.0)
Vascular health		
Myocardial infarction in 5 y, *n* (%)	1 (1.0)	4 (3.9)
Stroke in 5 y, *n* (%)	1 (1.0)	0
BMI, kg/m^2^	27.0 ± 5.6	27.5 ± 5.3
<18.5, *n* (%)	1 (1.0)	0
≥30, *n* (%)	18 (18.2)	25 (24.8)
Mini-Mental State Examination score	29 (28–29)^3^	29 (28–29)
Current prescription drugs,^4^ *n* (%)		
Statins	32 (38.1)	35 (44.9)
Proton-pump inhibitors	26 (31.0)	27 (34.6)
Other relevant	1 (1.2)	2 (2.6)
Dietary pattern, *n* (%)		
>1 portion meat/wk	69 (73.4)	70 (72.2)
>1 portion oily fish/wk	16 (18.0)	22 (23.4)
>1 portion white fish/wk	21 (21.9)	18 (18.8)
>1 portion eggs/wk	47 (48.5)	38 (39.2)
Daily alcohol	33 (34.4)	35 (35.4)
Blood biochemical measure		
Participants, *n*	86	84
Vitamin B-12, pmol/L	222.9 (197.4–268.9)	228.0 (194.7–271.0)
Holotranscobalamin, pmol/L	50.4 (38.2–68.3)	48.8 (39.8–62.9)
Homocysteine, μmol/L	15.9 (14.0–18.9)	16.3 (13.3–19.9)
Folate, nmol/L	17.7 (10.8–25.4)	17.5 (11.8–25.4)
Hemoglobin, g/L	139.8 ± 11.1	138.9 ± 12.9

1 OPEN, Older People and Enhanced Neurological Function.

2 Mean ± SD (all such values).

3 Median; IQR in parentheses (all such values).

4 Drug categories were as follows: statins (simvastatin, atorvastatin, pravastatin, and rosuvastatin); proton-pump inhibitors (omeprazole, lansoprazole, esomeprazole, rabeprazole, and pantoprazole); and other relevant drugs (amiodarone and metronidazole).

**TABLE 2:** Neurologic function at baseline by allocated treatment^1^

	Vitamin B-12 (*n* = 99)	Placebo (*n* = 100)^2^
Motor nerve conduction		
Posterior tibial CMAP amplitude (primary outcome), mV	4.6 (0–18.0)^3^	4.9 (0–13.6)
Posterior tibial conduction velocity,^4^ m/s	39.9 ± 5.0^5^	40.1 ± 5.2
Common peroneal CMAP amplitude, mV	2.2 (0–8.8)	2.5 (0–8.2)
Common peroneal conduction velocity,^4^ m/s	42.5 ± 4.6	43.0 ± 4.1
Sensory nerve conduction		
Sural SAP amplitude, μV	3.8 (0–17.5)	3.8 (0–14.2)
Sural conduction velocity,^6^ m/s	40.6 ± 5.2	40.2 ± 5.3
Superficial peroneal SAP amplitude, μV	2.4 (0–13.2)	3.4 (0–16.7)
Superficial peroneal conduction velocity,^7^ m/s	41.2 ± 6.0	41.0 ± 5.2
Central motor conduction		
Right abductor digiti minimi motor evoked potential amplitude, mV	3.3 ± 1.4	3.4 ± 1.5
Central motor conduction time, right abductor digiti minimi, ms	5.5 ± 1.2	5.5 ± 1.4
Central motor conduction time, right abductor hallucis,^4^ ms	13.6 ± 3.3	13.6 ± 3.5
Clinical nerve outcomes		
Absent right leg knee jerk, *n* (%)	11 (11.1)	8 (8.0)
Absent right leg ankle jerk, *n* (%)	33 (33.3)	22 (22.0)
Absent right great toe position sense, *n* (%)	4 (4.0)	8 (8.0)
Absent right great toe vibration sense, *n* (%)	66 (66.7)	66 (66.0)
Timed up-and-go, s	10.4 ± 3.0	10.7 ± 3.5

1 CMAP, compound muscle action potential; SAP, sensory action potential.

2 Two participants randomly assigned to placebo provided no baseline nerve function data.

3 Median; range in parentheses (all such values).

4 Small amounts of missing data (*n* < 10 in each arm).

5 Mean ± SD (all such values).

6 Missing data (*n* = 11 in vitamin B-12 and *n* = 16 in placebo).

7 Missing data (*n* = 23 in vitamin B-12 and *n* = 17 in placebo).

**TABLE 3:** Cognitive and psychological function at baseline by allocated treatment^1^

	Vitamin B-12 (*n* = 99)	Placebo (*n* = 102)
California Verbal Learning Test		
Total words correct in first 3 trials, *n*	22.8 ± 6.0	22.0 ± 6.5
Words recalled at delayed recall, *n*	7.3 ± 2.6	7.0 ± 3.1
Symbol letter modality,^2^ *n* correct	41.1 ± 9.5	39.6 ± 12.5
Reaction time, s		
Simple	0.3 ± 0.1	0.3 ± 0.1
Choice	0.7 ± 0.1	0.7 ± 0.2
Verbal fluency, *n* animals named	21.4 ± 5.4	21.3 ± 6.0
30-item General Health Questionnaire score^3^	2.5 ± 4.7	2.9 ± 4.7

1 All values are means ± SDs.

2 Missing data (*n* = 1 in vitamin B-12 and *n* = 1 in placebo).

3 Missing data (*n* = 8 in vitamin B-12 and *n* = 9 in placebo).

### Intervention participation

Participants were provided with 420 tablets over the course of the study and continued to receive the randomized treatment for an average of 389 d. There was no difference between trial arms in the number of tablets returned at the end of the study (mean: 37 tablets in the vitamin B-12 arm and 39 tablets in the placebo arm). The number of tablets apparently consumed closely matched the number of days in the study, which confirmed a very high level of adherence with allocated treatment (>97%). Blood samples were available from 151 participants at both baseline and 12 mo (**Table 4**). Allocation to vitamin B-12 was associated with a 177% increase compared with baseline in serum vitamin B-12 (641 compared with 231 pmol/L), a 331% increase compared with baseline in serum holotranscobalamin (240 compared with 56 pmol/L), and 17% lower serum homocysteine compared with baseline (14.2 compared with 17.1 μmol/L). In comparison, compared with baseline amounts, allocation to the placebo was associated with small changes (0–5%) in biochemical variables (Table 4).

**TABLE 4:** Effects of vitamin B-12 on serum concentrations of vitamin B-12, holotranscobalamin, homocysteine, folate, and hemoglobin^1^

	Vitamin B-12				Placebo			
Serum measure	*n*	Baseline	12 mo	Change from baseline, %	*n*	Baseline	12 mo	Change from baseline, %
Vitamin B-12, pmol/L	74	231.3 ± 52.0^2^	640.9 ± 199.3	177	70	235.4 ± 60.7	235.7 ± 77.5	0
Holotranscobalamin, pmol/L	71	55.7 ± 21.5	240.0 ± 162.9	331	70	51.8 ± 17.5	54.2 ± 29.5	5
Homocysteine, μmol/L	73	17.1 ± 4.6	14.2 ± 4.2	−17	70	17.2 ± 5.6	17.4 ± 6.0	1
Folate, nmol/L	72	20.7 ± 12.3	20.2 ± 11.6	−2	71	21.0 ± 13.8	20.4 ± 14.0	−3
Hemoglobin, g/L	78	140.5 ± 11.0	140.0 ± 10.7	0	71	137.9 ± 12.8	137.2 ± 12.6	0

1 Small amounts of missing data (*n* < 10/analyte).

2 Mean ± SD (all such values).

### Effects on neurologic function

Of participants allocated to the placebo arm, the nerve conduction test-retest correlation for the primary outcome of posterior tibial CMAP amplitude was 0.82, which showed a high level of reliability of nerve conduction measurements. Change in the primary outcome over the course of the study was small in both vitamin B-12 and placebo arms. There was no evidence of effect on the primary outcome by allocated treatment at 12 mo (mean difference: −0.2 mV; 95% CI: –0.8, 0.3 mV) or on any secondary peripheral nerve or central motor function outcome or on clinical examination (**Table 5**). Additional adjustment for age and sex did not alter these findings (Table 5).

**TABLE 5:** Effects of vitamin B-12 on the primary and secondary neurologic function outcomes at 12 mo^1^

	Vitamin B-12 (*n* = 91)	Placebo (*n* = 91)	Unadjusted effect size^2,3^	Adjusted effect size^3,4^
Motor nerve conduction				
Posterior tibial CMAP amplitude (primary outcome), mV	4.7 (0–15.3)^5^	5.3 (0–17.1)	−0.2 (−0.8, 0.3)	−0.2 (−0.9, 0.3)
Posterior tibial conduction velocity, m/s	39.1 ± 0.5^6^	40.2 ± 0.5	−0.7 (−2.0, 0.5)	−0.9 (−2.1, 0.6)
Common peroneal CMAP amplitude, mV	2.3 (0–8.0)	2.3 (0–6.6)	0.0 (−0.3, 0.3)	−0.0 (−0.3, 0.3)
Common peroneal conduction velocity, m/s	42.3 ± 0.5	43.1 ± 0.5	−0.4 (−1.3, 0.7)	−0.4 (−1.6, 0.6)
Sensory nerve conduction				
Sural SAP amplitude, μV	3.2 (0–18.7)	3.1 (0–18.5)	−0.6 (−1.5, 0.2)	−0.5 (−1.4, 0.3)
Sural conduction velocity,^7^ m/s	40.3 ± 0.5	40.9 ± 0.5	−1.0 (−2.2, 0.3)	−1.1 (−2.5, 0.1)
Superficial peroneal SAP amplitude, μV	3.1 (0–19.5)	3.2 (0–14.5)	0.1 (−0.7, 1.0)	0.1 (−0.7, 1.1)
Superficial peroneal conduction velocity,^8^ m/s	40.8 ± 0.6	40.8 ± 0.5	−0.6 (−2.3, 1.4)	−0.4 (−2.1, 1.2)
Central motor conduction				
Right abductor digiti minimi motor evoked potential amplitude, mV	3.5 ± 0.1	3.6 ± 0.1	0.0 (−0.3, 0.4)	0.0 (−0.3, 0.4)
Central motor conduction time, right abductor digiti minimi, ms	6.2 ± 0.1	6.2 ± 0.1	−0.0 (−0.4, 0.4)	−0.0 (−0.4, 0.4)
Central motor conduction time, right abductor hallucis,^9^ ms	14.0 ± 0.3	14.0 ± 0.3	0.1 (−0.8, 1.1)	0.1 (−0.8, 1.1)
Clinical nerve outcomes				
Absent right leg knee jerk, *n* (%)	14 (15.4)	9 (9.9)	1.2 (0.4, 3.7)^10^	1.1 (0.3, 3.6)^10^
Absent right leg ankle jerk, *n* (%)	33 (36.3)	22 (24.2)	0.8 (0.3, 2.0)^10^	0.8 (0.3, 2.0)^10^
Absent right great toe position sense, *n* (%)	4 (4.4)	4 (4.4)	1.4 (0.4, 5.1)^10^	1.4 (0.4, 5.1)^10^
Absent right great toe vibration sense, *n* (%)	57 (62.6)	52 (62.6)	0.8 (0.4, 1.4)^10^	0.8 (0.4, 1.4)^10^
Timed up-and-go, s	10.4 ± 2.6	10.7 ± 3.2	−0.12 (−0.6, 0.4)	−0.13 (−0.7, 0.4)

1 CMAP, compound muscle action potential; SAP, sensory action potential.

2 ANCOVA models were adjusted for baseline neurologic function.

3 Unless otherwise stated, all values are mean differences; 95% CIs in parentheses.

4 ANCOVA models were adjusted for baseline neurologic function, age, and sex.

5 Median; range in parentheses (all such values).

6 Mean ± SE (all such values).

7 Missing data (*n* = 19 in vitamin B-12 and *n* = 15 in placebo).

8 Missing data (*n* = 22 in vitamin B-12 and *n* = 22 in placebo).

9 Small amounts of missing data (*n* < 10 in each arm).

10 OR; 95% CI in parentheses.

### Effects on cognitive function and other secondary outcomes

The change in the main cognitive function outcome of the CVLT over the course of the study was small in both the vitamin B-12 and placebo arms. There was no evidence of an effect by allocated treatment on the CVLT at 12 mo (mean difference: −1.4 words; 95% CI: −2.9, 0.1 words) or on any other cognitive function outcome or psychological health (**Table 6**).

**TABLE 6:** Effects of vitamin B-12 on cognitive and psychological function outcomes at 12 mo

	Vitamin B-12 (*n* = 91)	Placebo (*n* = 93)	Unadjusted effect size^1^	Adjusted effect size^2^
California Verbal Learning Test				
Total words correct in first 3 trials, *n*	23.9 ± 0.7^3^	24.6 ± 0.7	−1.4 (−2.9, 0.1)^4^	−1.4 (−2.9, 0.1)
Words recalled at delayed recall, *n*	7.5 ± 0.3	7.7 ± 0.4	−0.4 (−1.0, 0.2)	−0.4 (−1.0, 0.2)
Symbol letter modality, *n* correct	39.6 ± 1.1	40.1 ± 1.2	−1.3 (−3.2, 0.6)	−1.3 (−3.2, 0.6)
Reaction time, s				
Simple	0.3 ± 0.01	0.3 ± 0.01	0.01 (−0.02, 0.04)	0.01 (−0.02, 0.04)
Choice	0.7 ± 0.01	0.7 ± 0.02	−0.003 (−0.03, 0.02)	−0.003 (−0.03, 0.02)
Verbal fluency, *n* animals named	20.8 ± 0.5	19.9 ± 0.6	1.1 (−0.1, 2.2)	1.1 (−0.1, 2.2)
30-item General Health Questionnaire score^5^	2.4 ± 0.5	2.7 ± 0.5	−0.1 (−1.2, 1.0)	−0.1 (−1.3, 1.1)

1 ANOVA models were adjusted for baseline cognitive function.

2 ANOVA models were adjusted for baseline cognitive function, age, and sex.

3 Mean ± SE (all such values).

4 Mean difference; 95% CI in parentheses (all such values).

5 Missing data (*n* = 5 in vitamin B-12 and *n* = 11 in placebo).

## DISCUSSION

We randomly assigned 201 nonanemic adults aged ≥75 y who had moderate vitamin B-12 deficiency to receive 1 mg vitamin B-12 or placebo/d for 12 mo. At baseline, participant characteristics, the primary outcome, and secondary neurologic and cognitive outcomes were well matched by allocated treatment groups, and loss to follow-up over 12 mo was <5%. The substantial changes in blood concentrations of vitamin B-12, holotranscobalamin, and homocysteine, in response to the allocated treatments showed a high level of adherence to the allocated study treatment over 12 mo. However, results of this trial showed no evidence of an effect on any measure of peripheral or central nerve conduction or of cognitive function by allocated treatment and did not provide any evidence that correction of moderate vitamin B-12 deficiency in the absence of anemia has beneficial effects on neurologic or cognitive function.

We identified trial participants with moderate vitamin B-12 status by using standard clinical testing procedures (serum vitamin B-12 concentration) (3). This identification was done to enhance the relevance of our trial for population health in older people although the appropriateness of various hematologic tests to assess vitamin B-12 status is currently under review (30). We selected neurologic and cognitive assessments for our trial that relate to strength, coordination, mobility, memory, processing speed, and executive function and are highly relevant to population health and quality of life in older people. Nerve conduction tests, in particular, provided objective measures of neurologic function by using state-of-the-art methods, and all testing was conducted by a single expert clinician both at baseline and study endpoint, which eliminated interobserver variability. We collected clinical measures of neurologic function (e.g., knee and ankle jerks and others) to support the relevance of the study. The study protocol was designed to minimize inconvenience and disturbance for participants, and we had high participant retention.

Study exclusion criteria resulted in the selection of relatively healthy and highly functioning participants who may have been less likely to benefit from vitamin B-12 supplementation. There are no age-specific reference data for neurologic function in older people (31), and norms for cognitive function (32, 33) are rarely population specific, which make interpretation problematic. It may be that neurologic and cognitive function was not impaired in study participants at baseline. However, this trial was designed to identify neurologic and cognitive benefits from vitamin B-12 supplementation in older people with moderate vitamin B-12 deficiency irrespective of baseline function. Because of the healthy nature of trial participants, the results may not be fully generalizable to all older people in the population. It is also possible that the duration of treatment may have been too short, and any effects of vitamin B-12 supplementation may only become evident after several years of supplementation or follow-up (34). No relevant trial with vitamin B-12 supplementation >2 y has been conducted to our knowledge (34). The dose of vitamin B-12 used in the study may have been insufficient. We selected a safe dose that was within current guidelines, and the intervention profoundly improved vitamin B-12 and holotranscobalamin status. Finally, the sample might have been too small adequately to detect a small change. The study had adequate power to detect a 28% change in the primary neurologic outcome with the assumption of a test-retest correlation of 0.6. Our test-retest correlation was 0.8, which suggested that we had power to detect a smaller change than was originally planned.

Observational studies have reported direct associations of vitamin B-12 status with nerve conduction (6), although this result has not been a consistent finding (7, 9, 35). To our knowledge, there has been no previous randomized controlled trial that assessed the impact of vitamin B-12 supplementation on neurologic function in older people. Previous trials of the effect of vitamin B-12 supplementation on cognitive function have largely shown no benefits of supplementation but have been of variable quality, small size, and short duration (10, 34). Some evidence of a benefit from multiple B-vitamin supplementation was recently reported, especially in subgroups of individuals with worse biochemical status when randomly assigned at baseline (36, 37). Our trial contributes robust evidence on the effect of vitamin B-12 on cognitive function in later life, and our findings are consistent with a recent meta-analysis that showed no effect of supplementation with vitamin B-12 on cognitive aging (38).

In conclusion, the current study did not detect any benefits of daily vitamin B-12 supplementation over 1 y on neurologic or cognitive function in asymptomatic, nonanemic older people with moderate vitamin B-12 deficiency. These results are directly relevant to current clinical practice, which identifies low vitamin B-12 status, especially in older people, as being a risk factor for neurologic and cognitive impairments. Moreover, the results of the current study cast doubt on the relevance of screening for moderate vitamin B-12 deficiency in the absence of anemia and symptoms of neurologic or cognitive impairment, suggesting the need for more-stringent definitions of vitamin B-12 deficiency (30). Our findings suggest that, with regard to peripheral motor and sensory nerve conduction, central motor conduction, and cognitive function, concerns over moderate vitamin B-12 deficiency in the absence of anemia in asymptomatic, older adults may not be justified.

## Footnotes

8 Abbreviations used: ADM, abductor digiti minimi; AH, abductor hallucis; CMAP, compound muscle action potential; CVLT, California Verbal Learning Test; MEP, motor evoked potential; SAP, sensory action potential.
